# Increasing risk of postlung transplant hospitalizations for infection: An analysis of recent trends

**DOI:** 10.1016/j.jhlto.2025.100231

**Published:** 2025-02-26

**Authors:** Shi Nan Feng, Armaan F. Akbar, Alice L. Zhou, Andrew Kalra, Sean Agbor-Enoh, Christian A. Merlo, Errol L. Bush

**Affiliations:** a Department of Surgery, Johns Hopkins University School of Medicine, Baltimore, Maryland; b Sidney Kimmel Medical College, Thomas Jefferson University, Philadelphia, Pennsylvania; c Department of Medicine, Johns Hopkins University School of Medicine, Baltimore, Maryland

**Keywords:** lung transplant, hospitalization, infection, trends, survival

## Abstract

**Background:**

Despite advancements in lung transplantation (LT), infection remains a major cause of morbidity and mortality following LT. We examined trends in hospitalizations for infection in the first year after LT.

**Methods:**

We identified adult LT recipients in the United States (March 1, 2018-March 9, 2023) using the Organ Procurement and Transplantation Network database. We categorized transplants into 3 eras to account for the Composite Allocation Score allocation policy change and coronavirus disease 2019: March 2018 to March 2020, March 2020 to March 2022, and March 2022 to March 9, 2023. One-year post-LT survival was compared using Kaplan-Meier survival analysis and Cox proportional hazards regression. Hospitalizations for infection were compared using multivariable logistic regression, adjusted for era and donor and recipient characteristics.

**Results:**

Of 12,388 LT recipients (median age = 62, male = 61.2%), hospitalization for infection in the first-year post transplant was 5.2% for patients transplanted from March 2018 to March 2020 (N = 5,031), 7.6% from March 2020 to March 2022 (N = 4,659), and 13.2% post-March 2022 (N = 3,640) (*p* < 0.001). Compared to March 2018 to March 2020, patients transplanted from March 2020 to March 2022 (adjusted aoods ratio [aOR] = 1.50, 95% confidence interval [CI] = 1.26-1.79) and post-March 2022 (aOR = 2.89, 95% CI = 2.29-3.65) were more likely to be hospitalized for an infection. After adjustment, we found no significant difference in risk of death following LT for recipients transplanted between March 2020 and March 2022 (aHR = 1.09, 95% CI = 0.96-1.23, *p* = 0.175) compared to March 2018 and March 2020. Post-March 2022 risk of death was elevated (aHR = 1.21, 95% CI = 1.04, 1.40, *p* = 0.014).

**Conclusions:**

Odds of hospitalization for infection in the first year after LT performed between March 2020 and March 2022 and post-March 2022 were 1.50 and 2.89 times as high, respectively, as LT performed between March 2018 and March 2020.

**IRB NUMBERS:**

IRB00352819

## Background

While lung transplantation (LT) has become the standard of care for patients with advanced lung disease of various etiologies, it remains a major operation with significant surgical risks, postoperative complications, and a median post-LT survival of only 6.7 years.^1^, ^2^ In particular, post-transplant infection, one of the most common transplant-related complications in LT recipients, is a major cause of morbidity and mortality following LT. In fact, LT recipients have lower survival rates compared to other solid organ transplant recipients due to higher rates of infection and rejection-related complications.^3^ Furthermore, infections are the second greatest cause of mortality within 30 days after LT.^4^ As such, the success LT often hinges on achieving a delicate balance between adequate immunosuppression to prevent rejection and minimizing the risk of post-transplant infection.

LT recipients are at a heightened risk of infection due to factors including immunosuppressive therapies to prevent organ rejection, continuous exposure of lungs to the environment, complications at the anastomotic sites, and impaired mucociliary clearance.^5^ Notably, both recipient and donor lung factors contribute to this increased risk. Pretransplant colonization of the recipient’s upper airways and proximal tracheobronchial tree by microorganisms, even in the absence of active infection, may serve as a reservoir that increases susceptibility to post-transplant infections in the setting of immunosuppression. Furthermore, the microbial burden and inflammatory status of donor lungs, which may vary depending on donor lung injury, aspiration events, or infections before procurement, can further predispose recipients to post-transplant infections. While individual risk factors for infection can include comorbidities such as diabetes and obesity, immune dysfunction can also be triggered and sustained by epidemiological factors such as community pathogens, the impact of viral pandemics, and other environmental factors that may alter the microbial milieu of LT recipients.^3^, ^6^, ^7^ While previous studies have characterized the prevalence and risk factors associated with specific types of post-transplant infections, none have elucidated recent trends in post-LT infectious complications.

Therefore, we sought to examine trends in hospitalizations for infection in the first year after LT by recent time eras, considering pre- and postcoronavirus disease 2019 (COVID-19) pandemic contexts. We hypothesize that our analysis will reveal rising post-LT infection risk in recent years, driven in part by the effects of the COVID-19 pandemic and ongoing challenges in the postpandemic period.

## Methods

### Data source

The data for this study were obtained from UNOS, serving as the contractor for the Organ Procurement and Transplantation Network (OPTN). The analysis and presentation of these data are the sole responsibility of the authors and should not be seen as an official position or interpretation by either the OPTN or the U.S. Government. Additionally, the Johns Hopkins Institutional Review Board has granted an exemption for this study from review (IRB00352819, approved on December 19, 2022). Furthermore, this study was conducted in compliance with the ethical principles outlined in the International Society for Heart and Lung Transplantation Ethics Statement. As this study utilized preexisting and deidentified data from the UNOS database, obtaining informed consent from individual participants was not required.

### Study population

Using Scientific Registry of Transplant Recipients (SRTR) data, we first identified all adult (≥18 years old) LT recipients without previous or multiorgan transplants between March 1, 2018, and March 9, 2023. The start and end dates for our study period were selected in consideration of pre-, during, and post-COVID-19 pandemic contexts, and the March 2023 Composite Allocation Score policy change. March 2018 was selected as the starting date to allow for 3 periods of approximately equal duration and comparable sample sizes. Accordingly, we further categorized transplants into 3 eras: March 1, 2018 to March 2020, March 2020 to March 2022, and March 2022 to March 9, 2023. The March 2022 end-point for the second era was selected based on observed reductions in COVID-19 infection, widespread easing of public health restrictions, and the transition to a postpandemic healthcare environment, providing a practical distinction between pandemic and postpandemic contexts. The first era, March 1, 2017 to March 2020, was used as the reference era.

### Statistical analysis

The normality of variables was assessed using Shapiro-Wilk testing and histogram visualization. Donor, recipient, and transplant characteristics of LTs performed by era were compared using chi-square testing for categorical variables and the Kruskal-Wallis test for continuous variables.

To compare hospitalizations for infection in the first-year post-LT by era, we performed multivariable logistic regression. We adjusted for relevant donor, recipient, and transplant characteristics. Covariates adjusted for in the final multivariable models were selected according to clinical relevance and by significance at a level of *p* < 0.1 on univariate analysis. Covariates included as follows: era, recipient age, recipient sex, recipient race, recipient body mass index, recipient diabetes, recipient extracorporeal membrane oxygenation (ECMO), recipient ventilator, recipient blood group, recipient days on waitlist, procedure type (bilateral vs single lung transplant), donor age, donor sex, donor race, induction therapy (antithymocyte globulins and basiliximab). The same covariates were used for adjustment in all multivariable models. All multivariable analyses were conducted as complete case analyses.

For post-transplant outcomes available in SRTR as binary variables, including acute rejection, prolonged ventilation (>48 hours), airway dehiscence, reintubation, post-transplant dialysis, and post-transplant stroke, we compared the proportion of recipients transplanted by era using chi-square or Fisher’s exact testing for univariate analysis and multivariable logistic regression for multivariate analysis. The covariates used in the multivariable models for all post-transplant outcomes are the same as above. Post-transplant length of stay was analyzed as a continuous variable, with the comparison of the distribution of length of stay between groups using Wilcoxon rank-sum testing. Due to the highly skewed nature of the length of stay data, we did not perform multivariable linear regression on the length of stay data.

To evaluate all cause, 1-year post-LT mortality, we performed time-to-event analysis and visualized the incidence of death using Kaplan-Meier curves. We used Cox regression to compare the time to death among recipients transplanted by era, adjusting for donor, recipient, and transplant characteristics, as described above. We followed recipients until the outcome of interest or administrative censorship. The linearity assumption was examined for variables included in both the logistic and Cox regression models. Cox regression models were evaluated for violations of the proportional hazards assumption using complementary log-log plots, smoothed hazard estimates, and global tests of proportional hazards on the basis of Schoenfeld residuals. No violations of the proportional hazards assumption were noted for the adjusted models.

In SRTR, mortality and graft loss are reported by individual transplant centers, and ascertainment is supplemented through linkage to the Social Security Master Death File (mortality) and the waiting list (graft loss).

All adjusted results are indicated with a lowercase “a” before the point estimate type (i.e., adjusted aoods ratio [aOR] for adjusted odds ratio).

All analyses were performed using Stata 17.0/MP for Windows (College Station, Texas).

## Results

### Study population

Of 12,388 patients who received LT (median age = 62, male = 61.2%), 5,031 patients were transplanted in era 1 (October 18, 2018-March 2020), 4,659 patients were transplanted in era 2 (March 2020-March 2022), and 3,640 patients were transplanted in era 3 (March 2022-June 2023) (Table 1).

**Table 1:** Lung Transplant Recipient and Transplant Characteristics by Era

Characteristic	Pre-March 2020 (*N* = 5,031)	March 2020-March 2022 (*N* = 4,659)	Post-March 2022 (*N* = 3,640)	*p*-value
*Recipient characteristics*				
Age	62 (54-67)	62 (55-67)	63 (56-68)	<0.001
Male sex	3,058 (60.8%)	2,871 (61.6%)	2,194 (60.3%)	<0.001
Race				<0.001
White	3,915 (77.8%)	3,388 (72.7%)	2,626 (72.1%)	
Black	476 (9.5%)	474 (10.2%)	316 (8.7%)	
Hispanic	485 (9.6%)	576 (12.4%)	524 14.4%)	
Other	155 (3.1%)	221 (4.7%)	174 (4.8%)	
BMI	26 (23-29)	27 (23-29)	26 (23-29)	<0.001
Diabetes	806	688	547	<0.001
Smoking	2,894 (57.5%)	2,615 (56.1%)	1,953 (53.7%)	<0.001
Recipient diagnosis				
Obstructive	1,221 (24.3%)	1,018 (21.9%)	728 (20.0%)	
Pulmonary vascular	277 (5.5%)	253 (5.4%)	194 (5.3%)	
CF and immunodeficiency	418 (8.3%)	89 (1.9%)	67 (1.8%)	
Restrictive	3,115 (61.9%)	3,299 (70.8%)	2,648 (72.8%)	
Other	0 (0.0%)	0 (0.0%)	2 (0.1%)	
Blood type				0.082
A	1,944 (38.6%)	1,769 (38.0%)	1,444 (39.7%)	
AB	208 (4.1%)	186 (4.0%)	128 (3.5%)	
B	569 (11.3%)	551 (11.8%)	422 (11.6%)	
O	2,310 (45.9%)	2,153 (46.2%)	1,646 (45.2%)	
Days on waitlist	46 (14-135)	32 (11-98)	32 (11-100)	<0.001
Lung Allocation Score	42 (36-57)	41 (36-58)	42 (36-56)	<0.001
FEV1%	40 (25-57)	43 (27-60)	45 (29-61)	<0.001
FVC%	47 (37-60)	49 (38-62)	49 (39-64)	<0.001
Waitlist ECMO	320 (6.4%)	413 (8.9%)	249 (6.8%)	<0.001
Waitlist MV support	245 (4.9%)	310 (6.7%)	175 (4.8%)	<0.001
Type of operation				<0.001
Bilateral	3,697 (73.5%)	3,654 (78.4%)	2,882 (79.2%)	
Single lung	1,334 (26.5%)	1,005 (21.6%)	758 (20.8%)	
Ischemic time (hours)	5 (4-6)	6 (5-7)	6 (5-8)	<0.001
Induction therapy				
ATG	53 (1.1%)	23 (0.5%)	19 (0.5%)	<0.001
BSX	3,572 (71.0%)	3,545 (76.1%)	3,006 (82.6%)	<0.001

Abbreviations: ATG, antithymocyte globulin; BMI, body mass index; BSX, basiliximab; ECMO, extracorporeal membrane oxygenation; FeV1, forced expiratory volume in 1 second; FVC, forced vital capacity; MV, mechanical ventilation.

Recipient pretransplant ECMO increased from 6.4% (*n* = 320) to 8.9% (*n* = 413) between eras 1 and 2, before decreasing to 6.8% (*n* = 249) in era 3 (*p* < 0.001). Pretransplant ventilator support followed a similar trend, increasing from 4.9% (*n* = 245) to 6.7% (*n* = 310) in era 2, before decreasing to 4.8% (*n* = 175) in era 3 (*p* < 0.001). Meanwhile, median days on the waitlist decreased throughout the study period, from 46 (IQR 14-135) days in era 1 to 32 (11-98) days in era 2 to 32 (11-100) days in era 3 (*p* < 0.001). Finally, with respect to immunosuppression regimens, antithymocyte use decreased from 1.1% (*n* = 53) in era 1 to 0.5% in eras 2 (*n* = 23) and 3 (*n* = 19) (*p* < 0.001). Conversely, basiliximab use increased from 71.0% (n = 3,572) in era 1, to 76.1% (*n* = 3,545) in era 2, to 82.6% (*n* = 3,006) in era 3 (*p* < 0.001) (Table 1).

Donor age and BMI remained relatively consistent over the study period. Donor cause of death of anoxia increased across eras (33.5% [*n* = 1,685] in era 1 vs 35.9% [*n* = 1,673] in era 2 vs 40.4% [*n* = 1,472] in era 3), while stroke and central nervous system tumor decreased. Additionally, donation after circulatory death increased from 5.9% (*n* = 296) in era 1 to 7.6% (*n* = 355) in era 2 to 8.6% (*n* = 314) in era 3 (Table S1).

### Hospitalizations for infection

Of 685 patients hospitalized for infection in their first-year post-LT over the study period, the hospitalization rate was 5.2% (*n* = 245) for patients transplanted in era 1, 7.6% (*n* = 317) for patients transplanted in era 2%, and 13.2% (*n* = 123) for patients transplanted in era 3 (*p* < 0.001) (Table 2).

**Table 2:** Unadjusted Lung Transplant Outcomes by Era

Outcome	Pre-March 2020 (N = 3,645)	March 2020-March 2022 (N = 5,658)	Post-March 2022 (N = 4,360)	*p*-value
Hospitalization for infection	245 (5.2%)	317 (7.6%)	123 (13.2%)	<0.001
Acute rejection	411 (8.2%)	286 (5.1%)	152 (3.5%)	<0.001
Reintubation	981 (26.9%)	834 (14.7%)	538 (12.3%)	<0.001
Airway dehiscence	80 (2.2%)	71 (1.5%)	48 (1.8%)	0.002
Tracheostomy	199 (4.0%)	231 (5.0%)	119 (4.4%)	0.057
ECMO at 72 h	408 (8.1%)	485 (10.4%)	272 (10.1%)	<0.001
MV support	2,003 (40.0%)	1,932 (41.9%)	1,173 (44.0%)	0.002
Stroke	131 (2.6%)	129 (2.8%)	65 (2.4%)	0.64
Dialysis	444 (8.8%)	389 (8.3%)	255 (9.5%)	0.27
Hospital LOS (days)	19 (13-32)	19 (13-33)	20 (14-34)	<0.001

Abbreviations: ECMO, extracorporeal membrane oxygenation; LOS, length of stay; MV, mechanical ventilation.

Multivariable regression analyses showed that, compared to patients transplanted in era 1, odds of hospitalization for infection in the first year after LT were 1.50 times as high for patients transplanted during era 2 (aOR = 1.50, 95% confidence interval [CI] = 1.26-1.79, *p* < 0.001), and 2.89 times as high for transplants performed during era 3 (aOR = 2.89, 95% CI = 2.29-3.65, *p* < 0.001) (Table 3).

**Table 3:** Multivariable Analyses for Hospitalization for Infection in the First Year After Lung Transplant by Era

Outcome	Odds ratio	95% CI	*p*-value
Hospitalization for infection (ref: March 2018-March 2020)			
March 2020-March 2022	1.50	1.26, 1.79	<0.001
Post-March 2022	2.89	2.29, 3.65	<0.001

Adjusted for era, recipient age, recipient sex, recipient race, recipient body mass index, recipient diabetes, recipient extracorporeal membrane oxygenation, recipient ventilator, recipient blood group, recipient days on waitlist, donor age, donor sex, donor race, induction therapy (ATG/BSX)

### Recipient postoperative outcomes

Acute rejection in LT recipients decreased over the study period, from 8.2% (*n* = 411) in era 1, to 6.1% (*n* = 286) in era 2, to 5.8% (*n* = 208) in era 3 (*p* < 0.001) (Table 2) (era 2: aOR 0.75, 0.64-0.88, *p* < 0.001; era 3: aOR 0.73, 0.60, 0.89, *p* = 0.002) (Table 4). Rate of post-LT airway dehiscence (2.2% [*n* = 80] in era 1 vs 1.5% [*n* = 71] in era 2 vs 1.8% [*n* = 48] in era 3, *p* = 0.002) and reintubation (26.9% [*n* = 981] in era 1 vs 14.7% in era 2 [*n* = 834] vs 78.8% [*n* = 2,118] in era 3, *p* < 0.001) also decreased over the study period (Table 2), though odds between eras were similar after multivariate adjustment (Table 4).

**Table 4:** Multivariable Analyses for Lung Transplant Outcomes by Era

Outcome (ref: March 2018-March 2020)	Odds ratio	95% CI	*p*-value
Acute rejection			
March 2020-March 2022	0.75	0.64, 0.88	<0.001
Post-March 2022	0.73	0.60, 0.89	0.002
Reintubation			
March 2020-March 2022	0.90	0.81, 1.00	0.054
Post-March 2022	1.03	0.92, 1.17	0.582
Airway Dehiscence			
March 2020-March 2022	0.94	0.68, 1.30	0.716
Post-March 2022	1.13	0.78, 1.63	0.517
Tracheostomy			
March 2020-March 2022	0.97	0.74, 1.25	0.795
Post-March 2022	1.23	0.91, 1.66	0.180
ECMO at 72 h			
March 2020-March 2022	1.23	1.06, 1.42	0.005
Post-March 2022	1.28	1.08, 1.51	0.005
MV support			
March 2020-March 2022	1.05	0.96, 1.14	0.316
Post-March 2022	1.20	1.09, 1.33	<0.001
Stroke			
March 2020-March 2022	1.13	0.88, 1.45	0.342
Post-March 2022	0.98	0.72, 1.34	0.915
Dialysis			
March 2020-March 2022	0.91	0.78, 1.05	0.181
Post-March 2022	1.11	0.94, 1.31	0.209

Abbreviation: MV, mechanical ventilation.

Adjusted for era, recipient age, recipient sex, recipient race, recipient body mass index, recipient diabetes, recipient extracorporeal membrane oxygenation, recipient ventilator, recipient blood group, recipient days on waitlist, donor age, donor sex, donor race, induction therapy (ATG/BSX).

Conversely, the rate of post-transplant prolonged ventilator support increased from 40.0% (*n* = 2,003) in era 1, to 41.9% (1,932) in era 2, to 44.5% (1,592) in era 3 (*p* < 0.001) on univariate analysis (Table 2). Multivariate analysis was not statistically significant for patients receiving LT in era 2 compared to era 1 (aOR 1.05, 0.96-1.14, *p* = 0.316), but odds of prolonged ventilator support were 1.20 times as high in era 3 compared to era 1 (aOR 1.20, 1.09-1.33, *p* < 0.001) (Table 4). Post-transplant ECMO also had an overall increase, from 8.1% (*n* = 408) in era 1, to 10.4% (*n* = 485) in era 2, to 10.1% (*n* = 367) in era 3 (*p* < 0.001) (Table 2) (era 2: aOR 1.23, 1.06-1.42, *p* = 0.004; era 3: aOR 1.28, 1.08-1.51, *p* = 0.005) (Table 4). Post-transplant dialysis also increased over the study period, from 8.8% (n = 444) in era 1 to 9.1% (*n* = 332) in era 3 (*p* < 0.001) (Table 2), though multivariate analysis showed no statistical significance (aOR 1.11, 0.94-1.31, *p* = 0.209) (Table 4).

The rate of tracheostomy initially increased from 4.0% (*n* = 199) in era 1 to 5.0% (*n* = 231) in era 2, before decreasing to 4.1% (*n* = 150) in era 3. A similar trend was also seen in the rate of post-transplant stroke, from 2.6% (*n* = 131) in era 1, to 2.8% (*n* = 129) in era 2, followed by 2.4% (*n* = 88) in era 3 (*p* = 0.006) (Table 2). However, the differences in post-transplant tracheostomy and stroke did not persist after multivariable adjustment (Table 4). Finally, the median length of hospital stay had a slight increase over the study period, from 19 days (interquartile range, IQR, 13-32) to 20 days (IQR 14-33) (*p* < 0.001) (Table 2).

**Figure 1:**
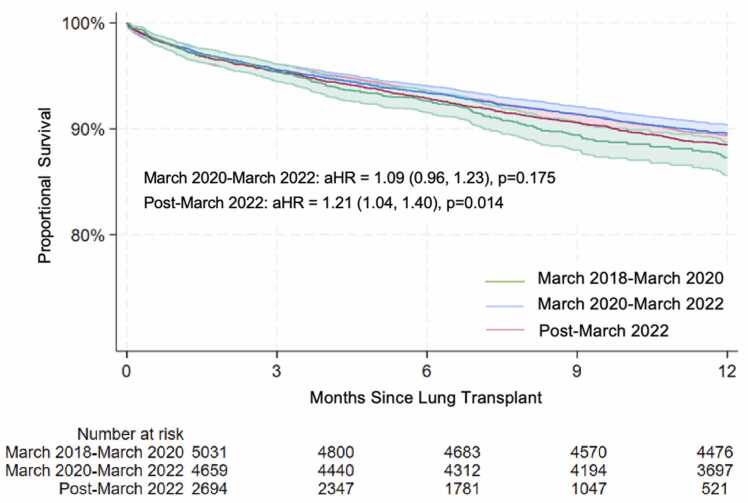
One-year postlung transplant survival by era.

After adjusting for donor and recipient characteristics, 1-year survival analysis showed no significant difference in the risk of death for recipients transplanted between March 2020 and March 2022 (aHR = 1.09, 95% CI = 0.96-1.23, *p* = 0.175) compared to March 2018 and March 2020. However, risk of death for recipients transplanted post-March 2022 was significantly elevated in comparison (aHR = 1.21, 95% CI = 1.04, 1.40, *p* = 0.014) (Figure 1).

## Discussion

In this national retrospective analysis of LT recipient outcomes, we found that patients who received LT between March 2020 and March 2022 had 1.50 times the odds of being hospitalized for infection within the first-year post transplant compared to those who received LT between March 2018 and March 2020. Furthermore, patients who received a transplant after March 2022 had 2.89 times the odds of being hospitalized for infection within the first-year post transplant compared to those transplanted between March 2018 and March 2020.

Our finding that both pretransplant ECMO and prolonged ventilator support increased in era 2 and then decreased in era 3 coincides with the peak of COVID-19 during era 2.^8^, ^9^ Meanwhile, we found that odds of hospitalizations for infection increased dramatically throughout the entire study period. This rise could be attributed to multiple factors related to both the COVID-19 pandemic and ongoing challenges in the postpandemic period. In addition to the risk of COVID-19 infection in LT recipients itself,^10^ the pandemic strained healthcare resources and personnel,^11^, ^12^ likely influencing perioperative and postoperative care for transplants performed during that time. Notably, previous studies have found both inadequate hospital infrastructure and resource and workforce shortages to be barriers precluding effective infection prevention and control in healthcare settings.^13^, ^14^, ^15^ Furthermore, a recent study of LT recipients receiving severe acute respiratory syndrome coronavirus 2 (SARS-CoV-2) mRNA vaccination found that LT recipients experienced diminished antibody response following vaccination compared to the general population; thus, reduced clinical efficacy of the vaccine in the LT recipient population which may also have contributed to rising infections in recent years.^16^ Interestingly, odds of hospitalization for infection continued to increase for transplants performed in era 3, despite the waning severity of COVID-19 after era 2.^17^ While we were unable to determine whether COVID-19 itself contributed to this trend, the persistent increase in infection-related hospitalizations may reflect long-term or enduring effects of the pandemic including residual immune system compromise,^18^ ongoing resource limitations, shifts in post-transplant care practices, and evolving infection prevention strategies in response to pandemic-era challenges. The possibility of residual effects arising from the COVID-19 pandemic context might also relate to our finding that patients receiving LT in era 3 had the highest odds of post-transplant prolonged ventilator support and ECMO. These factors could also partially explain the increased risk of death for patients receiving LT in era 3 compared to patients receiving LT in either eras 1 or 2.

We also discovered a significant decline in odds of acute rejection across the study period, despite the increased likelihood of other complications. The decline in the odds of acute rejection might reflect improvements in immunosuppressive therapy protocols or advancements in post-transplant care contributing to better management of graft rejection over time. Additionally, the modest difference in hospital length of stay between eras despite increasing ECMO and prolonged ventilation may be attributed to improvements in perioperative and critical care management, leading to earlier weaning from organ support. However, the rise in other complications, including infections, may suggest a shift in the risk profile for transplant recipients. The transition to the Composite Allocation Score system in 2023, which replaced the Lung Allocation Score, introduced broader considerations for candidate prioritization that may have influenced the types of patients receiving transplants and, consequently, post-transplant outcomes.^19^ Additionally, the increasing use of ex vivo lung perfusion and novel preservation systems has expanded donor lung utilization and may have altered post-transplant risk profiles as well.^20^ To this end, our findings highlight the complex balance between preventing rejection and managing infection in LT recipients, while also underscoring a pressing need to better understand and mitigate the influence of evolving allocation policies, preservation techniques, and future pandemics on post-transplant outcomes.

Our results are limited by the retrospective nature of our study design, and the data available in the UNOS database. While we were able to identify trends in post-LT hospitalizations for infection across Eras, we were unable to fully explore the reasons underlying these trends given the limitations of the registry data. Specifically, we were unable to capture detailed clinical data on the nature, causes, and timing of these hospitalizations, which limits our ability to draw definitive conclusions about the drivers of increased infection risk. As a result, while our findings clearly demonstrate an increased trend in hospitalizations after LT across the study periods, the underlying mechanisms remain speculative. Additionally, due to limitations of the registry data, we were unable to measure the time between transplant and hospitalization for infection. We were also unable to distinguish the type of infection, limiting the granularity of our study. Similarly, we were unable to determine whether recipients were infected with COVID-19 during any period of time. Finally, while the OPTN database provides generalizable results, we recognize that it does not include all transplant center, provider, and patient characteristics, which can result in unmeasured confounding.

In conclusion, we found an increasing risk of hospitalization for infection in the first year after LT performed in Era 2 compared to Era 1, and in Era 3 compared to both Eras 1 and 2. The relative risk of death was similar between Eras 1 and 2, but higher for transplants performed in Era 3. Our findings demonstrate an alarming rise in post-LT infections, indicating a profound need to better understand the mechanisms underlying these trends to improve patient care and outcomes for LT recipients. Additionally, our results highlight a critical need for more granular data and further research on the types and causes of infections in LT recipients, COVID-19-specific outcomes, and their impact on post-transplant hospitalizations and survival.

## GLOSSARY OF ABBREVIATIONS: (in order of appearance)

LT: lung transplant
COVID-19: Coronavirus disease 2019
UNOS: United Network for Organ Sharing
OPTN: Organ Procurement and Transplantation Network
SRTR: Scientific Registry of Transplant Recipients
ECMO: extracorporeal membrane oxygenation
LAS: lung allocation score
IQR: interquartile range
OR: odds ratio
aOR: adjusted odds ratio
SARS-CoV-2: Severe Acute Respiratory Syndrome Coronavirus 2
