# Supplementary material for: Increasing risk of postlung transplant hospitalizations for infection: An analysis of recent trends
Source: JHLT Open. 2025 Feb 26;8:100231. doi: 10.1016/j.jhlto.2025.100231 (PMC11935489; doi:10.1016/j.jhlto.2025.100231)
Supplement: Supplementary file 1 — Supplementary material [file mmc1.docx]

**SUPPLEMENTAL TABLE 1. Lung Transplant Donor Characteristics by Era.**

| **Characteristic** | **March 2018 to March 2020 (N=5,031)** | **March 2020 to March 2022 (N=4,659)** | **Post March 2022 (N=2,698)** | **p-value** |  |
| --- | --- | --- | --- | --- | --- |
| **Donor Characteristics** |  |  |  |  |  |
| Donor Age (years) | 34 (25-47) | 34 (25-46) | 35 (26-46) | <0.001 |  |
| Donor Male Sex | 3,049 (60.6%) | 2,849 (61.2%) | 2,174 (59.7%) | 0.58 |  |
| Donor Race |  |  |  | <0.001 |  |
| White | 3,152 (62.7%) | 2,704 (58.0%) | 2,147 (59.0%) |  |  |
| Black | 820 (16.3%) | 873 (18.7%) | 642 (17.6%) |  |  |
| Hispanic | 842 (16.7%) | 885 (19.0%) | 691 (19.0%) |  |  |
| Other | 217 (4.3%) | 197 (4.2%) | 160 (4.4%) |  |  |
| Donor BMI (kg/m^2^) | | 26 (23-30) | 26 (23-30) | 26 (23-30) | <0.001 |
| Smoking History | | 400 (8.0%) | 324 (7.0%) | 304 (8.4%) | <0.001 |
| Donor Cause of Death |  |  |  | <0.001 |  |
| Anoxia | 1,685 (33.5%) | 1,673 (35.9%) | 1,472 (40.4%) |  |  |
| Cerebrovascular/Stroke | 1,341 (26.7%) | 1,125 (24.1%) | 898 (24.7%) |  |  |
| Head Trauma | 30 (0.6%) | 19 (0.4%) | 14 (0.4%) |  |  |
| Central Nervous System Tumor | 1,875 (37.3%) | 1,748 (37.5%) | 1,181 (32.4%) |  |  |
| Other | 100 (2.0%) | 94 (2.0%) | 75 (2.1%) |  |  |
| Donation After Circulatory Death | 296 (5.9%) | 355 (7.6%) | 314 (8.6%) | <0.001 |  |
